# Tribological Behavior of Bioinspired Surfaces

**DOI:** 10.3390/biomimetics8010062

**Published:** 2023-02-02

**Authors:** Sachin Kumar Sharma, Harpreet Singh Grewal

**Affiliations:** Surface Science and Tribology Lab, Department of Mechanical Engineering, Shiv Nadar Institution of Eminence, Gautam Buddha Nagar 201314, Uttar Pradesh, India

**Keywords:** bioinspired, surface engineering solutions, tribological behavior, anti-wear surface, anti-adhesion surfaces, friction

## Abstract

Energy losses due to various tribological phenomena pose a significant challenge to sustainable development. These energy losses also contribute toward increased emissions of greenhouse gases. Various attempts have been made to reduce energy consumption through the use of various surface engineering solutions. The bioinspired surfaces can provide a sustainable solution to address these tribological challenges by minimizing friction and wear. The current study majorly focuses on the recent advancements in the tribological behavior of bioinspired surfaces and bio-inspired materials. The miniaturization of technological devices has increased the need to understand micro- and nano-scale tribological behavior, which could significantly reduce energy wastage and material degradation. Integrating advanced research methods is crucial in developing new aspects of structures and characteristics of biological materials. Depending upon the interaction of the species with the surrounding, the present study is divided into segments depicting the tribological behavior of the biological surfaces inspired by animals and plants. The mimicking of bio-inspired surfaces resulted in significant noise, friction, and drag reduction, promoting the development of anti-wear and anti-adhesion surfaces. Along with the reduction in friction through the bioinspired surface, a few studies providing evidence for the enhancement in the frictional properties were also depicted.

## 1. Introduction

Nature exhibits outstanding evolutionary abilities [1,2,3,4]. It has developed quite diverse and complex structures. Based on its evolutionary characteristics, nature has developed optimal solutions to adapt the different life forms to their local environment. Mimicking nature helps in solving many complex problems [5,6]. The foremost example of biomimicry is probably the design of flying machines by Leonardo da Vinci inspired by birds [7]. Although there are numerous instances, one of the most exciting areas where biomimicry has made a substantial contribution is the creation of superhydrophobic surfaces [8,9]. To obtain functional characteristics such as self-cleaning, non-wettable, anti-icing surfaces, lowering drag in submarines and other vessels, and for the self-propulsion of liquids in micro-channels, superhydrophobicity is necessary [10]. In addition to low adhesion and friction characteristics offered by superhydrophobic materials, nature has also devised other modulation strategies such as SLIPS (Slippery Liquid-infused Porous Surfaces), and anti-wear surfaces for sustaining extreme tribological challenges [11]. Superhydrophobic surface designs have been influenced by the surface structure of plants and insects, such as the lotus leaf, rose petal, and water strider’s feet [12,13].

**Figure 1:**
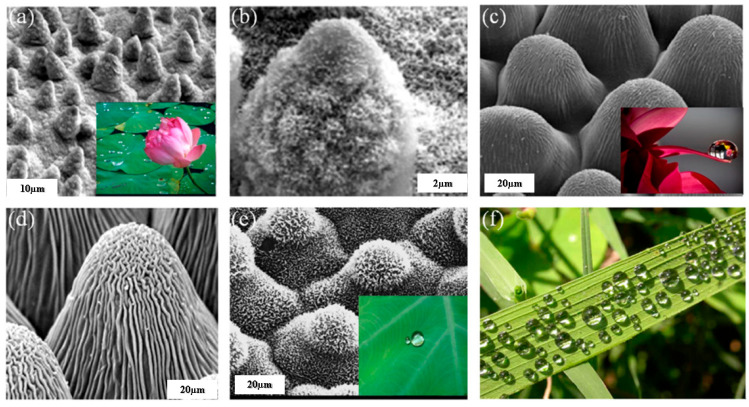
(**a**) SEM image of a lotus leaf with inset entailing the water droplet on the lotus leaf, (**b**) High magnification image of lotus leaf depicting the hierarchical structures, (**c**) SEM image of cuticles on the papillae of a dahlia with inset depicting the droplet on dahlia petal, (**d**) SEM image of cuticles on papillae of rose petal, (**e**) Epicuticular wax platelets over the taro leaf with inset depicting the droplet over the leaf, (**f**) Water droplets over the rice leaf entailing the super-hydrophobicity [1]. Copyright permission from IOP, 2015.

Figure 1 shows the hierarchical structures that make up such biological surfaces. These structures’ ability to repel water is enhanced by their hierarchy. Both the form and the length scale exhibit the hierarchy [14]. As seen in Figure 1a,b,e, the leaves of the taro (Colocasia esculenta) and the lotus, respectively, are composed of nanoscale wax structures with the shapes of platelets and tubules that are overlaid on papillae epidermal cells [1]. Similar to this, the papillae epidermal cells of the leaves of the Asteraceae plant family and the petals of roses and dahlias have overlaid cuticles depicted in Figure 1c,d. Water droplets passing over the rice leaf demonstrating the super-hydrophobicity are shown in Figure 1f. In the case of a superhydrophobic surface, the extreme water-repellent state is formed owing to the effective entrapment of air, contributing to lower friction and adhesion [15]. A composite air-solid interface modulates the surface interactions promoting droplet mobility [16]. Such tribological interactions are essential in many technical applications, such as the aerospace, marine, and textile industries [17]. The lotus leaves are the most relevant example of superhydrophobic surfaces owing to their self-cleaning and ultra-low-adhesion [18,19,20]. Due to low adhesion and friction, bioinspired surface morphology improves the tribological performance of the surfaces. 

The global energy dilemma of the twenty-first century is an increasingly critical issue. Numerous forms of transportation utilize a significant amount of energy. A major fraction of this energy is used to overcome friction [21]. Conventional ships and aircraft have a surface friction resistance of around 50% of the total resistance. Additionally, most of the pumping station’s power is utilized to overcome surface friction throughout the long-distance pipeline conveyance operation. Energy significantly restricts underwater robots’ operational range and duration [22]. Research on bio-inspired drag reduction has been the priority for energy saving since 1970 [23,24]. Nature offers numerous sources of inspiration which can be employed effectively for sustainable future advancement. For instance, fast-swimming sharks have special micro-grooves in their skin to aid reduction in friction [25]; the surface of a lotus leaf exhibits a water-repellent effect [26]; gecko feet have a smart-adhesion function that allows them to climb even the smoothest surfaces [27]. It is well acknowledged that friction is reduced with surface smoothness, but an investigation in 1982 revealed that shark skin has a micro-groove structure that can significantly minimize friction in some turbulent situations [28]. The rib pattern of shark skin is efficient for drag reduction [29]. Numerous advancements in biomimetic drag reduction have been made and can be categorized into three groups: non-smooth surfaces [30], surfaces that are highly hydrophobic [31,32], and surfaces that use water jets [23]. Table 1 depicts the various biomimetic surfaces utilized for drag reduction. The drag reduction by bionic surfaces is a prerequisite in conserving energy through air, entailing surface area reduction. Similar characteristics are depicted in the turtle body, persisting to drag reduction [33]. Furthermore, the modifications in the surface morphology and topography of bioinspired surfaces are also contributing towards a further reduction in friction, which saves energy consumption. 

The morphological modulation of the surface is an intriguing approach that finds immense use in the field of tribology [53]. For lubricated contacts, this strategy has proven to be quite successful; for instance, a reduction in friction by over 80% was achievable for a unidirectional steel-on-steel contact with circular dimples [54]. Attempts have been made to generate a bio-inspired surface morphology and understand its potential to reduce the friction forces in both lubricated and unlubricated interfaces. Different strategies for translating biological solutions have been created. To retain the essence of the biological solution holding the outcome of a protracted evolutionary adaptation process, great caution is required [55]. Nature offers a wide range of low-friction surfaces as an alternative [56,57]. Researchers emphasize the skin of several reptiles such as sandfish skink, restoring the anti-friction and anti-wear characteristics owing to their intense interactions with the land during locomotion [58,59]. More importantly, the surface morphology, along with the species scale and the location of scale, is of significant concern on the snake’s body that needs to be addressed while mimicking the snake-inspired surface [60]. The literature study depicted that the individual scales that make up a snake’s skin overlap each other and have frequent protrusions in the shape of teeth to reduce wear and friction [60,61,62]. However, the role of such scale-like surface topographies on metal surfaces in lowering the friction forces due to changes in structural stiffness and whether or not this is true for lubricated interactions has not been explored yet. Some bio-inspired approaches have been studied, and interesting results for polymer surfaces have recently been realized [53,63,64,65]. The ventral scales of the snake Phyton regius and the sand skink lizard served as inspiration for developing the surface morphologies promoting the reduction in friction [66]. Both creatures exhibit surface patterns with varying sizes as well as the usual scale-like pattern found on the skin [61]. The skin of sandfish has been extensively studied and is renowned for its low friction and high resistance to wear against the sand [30,53,67,68]. This characteristic has been used to produce a surface with a strong resistance to wear. In terms of low friction and high wear resistance, the micro- and nano-scale over the hierarchical pattern were also considered to be a beneficial methodology [69,70].

At the nano and micro-scales, the surface area to volume ratio considerably increases, which causes surface forces to have a high impact on the functionality of nano and micro-scale structures [71]. The intermolecular forces of the interacting phases define the final surface forces [72]. These intermolecular forces govern the tribological (friction and adhesion) and wetting behavior of the micro- and nano-scale systems [73]. Low friction and adhesion enhance many micro- and nano-electromechanical systems (MEMS/NEMS) endurance and effectiveness. It is typically advisable to employ low surface energy materials and texturing to reduce adhesion and friction between interacting surfaces. Numerous textural geometrical shapes that were inspired by nature have been used to significantly improve the tribological behavior of MEMS/NEMS [74,75]. Hierarchical patterns have demonstrated superior performance compared to their micro- and nano-counterparts [73,76,77]. Understanding the function of the micro- and nano-scalar aspects of the hierarchical patterns in the tribological and wetting behaviors is necessary to achieve superior performance [73]. The surface chemistry and different geometric parameters, pitch (distance between the pillars), height, and diameter of the micro- and nano-features are the factors defining the performance evaluation of different surface textures/patterns [78]. The tribological and wetting behavior of these features is also influenced by their shape [79]. The link between geometric factors and friction and adhesion is highly complex, in contrast to surface chemistry [80]. The mechanical reliability of the pattern geometry is somewhat responsible for this intricate relationship. High stresses can cause deformation, which can relate to the patterns’ erratic behavior. The level of deformation for a particular pattern depends on the material and geometrical characteristics [81]. 

**Figure 2:**
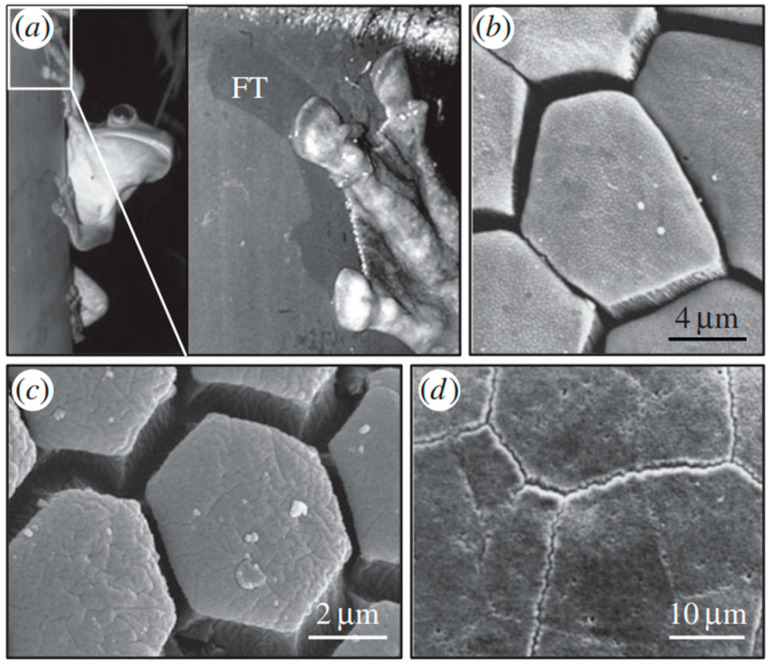
(**a**) On a tree trunk, a rusted tree frog. Due to the limb sliding before friction stopped the action, fluid secretion traces were left on the surface of the tree (FT), and the surface pattern was obtained in (**b**) tree frog, (**c**) bush cricket, and (**d**) mushroom-tongued salamander [89]. Copyright Permission 2014, Royal Society.

Many studies on hairy attachment mechanisms have led to a new field of study addressing the gecko adhesion effect [82]. Contrarily, smooth attachment methods have gotten much less attention, which begs for more research given their equally remarkable characteristics, such as excellent resistance to slippage. Focusing on the smooth contact pads that amphibians, insects, and mammals have developed to improve the ability of their feet to cling to objects can lead to exciting applications [83]. The research studies depicted that such surfaces have different surface micropatterns that act in the presence of fluid secretion, such as an oil-in-water emulsion in the case of insects [84]. Additionally, some of the animals with lubricated pads with smooth patterns jump, which involves a lot of friction when pushing off and landing [85]. The contact pads of these creatures have one of the most stunning surface textures ever seen. It is based on a hexagonal pattern that originated in bush crickets, tree and torrent frogs, and mushroom-tongued salamanders, as shown in Figure 2a–d. The hexagonal surface pattern was recognized as a friction-oriented characteristic capable of decreasing stick-slip and hydroplaning while enabling friction adjustment [86]. Besides these hexagonal structures, bioinspired bio-materials also paved the way for developing low-friction surfaces [87,88]. Dopamine is among the category of green oil-soluble additives, providing low-friction have been discussed in detail in the work-study. 

Modern bio-inspired functional materials can be designed for solid particle erosion resistance. For instance, the body coverings of the scorpion and tamarisk, which have a unique surface structure that is present everywhere, can withstand sand erosion very well [90]. By altering solid particle erosion parameters, such structure can increase the resistance of naturally created surfaces against solid particle erosion [91]. The cuticle of a lobster and the nacre of a shell have unique interior structures. Because the unique interior structure can increase the fracture toughness of the natural materials, the nacre is employed for safeguarding internal soft tissue and the cuticle [92]. The two-layer structure, which includes a hard layer and a soft layer, has a buffering effect, which helps the skin of desert lizards and sandfish endure wind-blown sand quite effectively [78,93]. The internal vascular system of the skin or bone can provide healing agents to injury sites for self-healing to repair the damage and mitigate further damage [94]. Therefore, precise and accurate solutions to solid particle erosion could be developed by mimicking natural materials with these unique architectures. Furthermore, surface texturing was also considered a beneficial aspect of strengthening the surface properties of the bioinspired surfaces [54,95,96,97,98]. 

Surface textures like dimples, grooves, or convex on surfaces of friction units using mechanical or chemical processing technologies, have gained popularity as a means to enhance the tribological performance of mechanics [99]. Surface texturing has been used extensively in engineering since the idea of fabricating microstructures on mechanical friction pairs as textures was first proposed in the 1960s. Examples include minimizing frictional resistance and side leakage by arranging textures on mechanical seals and reducing abrasion and energy consumption by manufacturing micro-grooves on automobile piston rings [100]. Recent decades have seen an increase in the development of surface texturing techniques to enhance material performance and features, which can be attributed to the rise in the demand for materials in various applications. Due to its capacity to regulate exterior qualities in specific applications, such as self-cleaning of surfaces in medicine and anti-biofouling, surface texturing has emerged as a crucial field in material science [94,101]. By carefully evaluating the effect of texturing on materials under various tribological conditions, including cavitation wear, adhesive wear, and wear with lubrication, numerous studies showed improvement in tribological performance. Significant studies explored surface texturing to address the need to improve tribological characteristics like wear and friction. Surface texturing is frequently used to improve the mechanical characteristics of segments [102]. It was discovered that surface texturing could enhance not just tribological characteristics but also light absorption in solar cells, the performance of biological implants, and the ability to create super-hydrophobic coatings. Laser surface texturing can dramatically increase the wettability of materials [103]. Improved lubricating coatings can result in super-hydrophobic coatings attributed to surface texturing. Surface texturing involves forming, micro-grooving, micro-dimples, and microchannels, among other surface modifications [104]. During the process of making the material, surface textures can be created. Inverted pyramids, micro-dimples, micro-grooves, nano-dots, micro-pits, and other surface texturing structures have all been created and studied [103]. For tribological applications, research was done on textured surfaces of various sizes and shapes. Additionally, it has been investigated how different surface texturing characteristics, such as spacing, dimensions, geometries, distance, width, area fractions, and tuning the depth of the micro/nanostructures, affect tribological properties [103,105,106,107].

**Figure 3:**
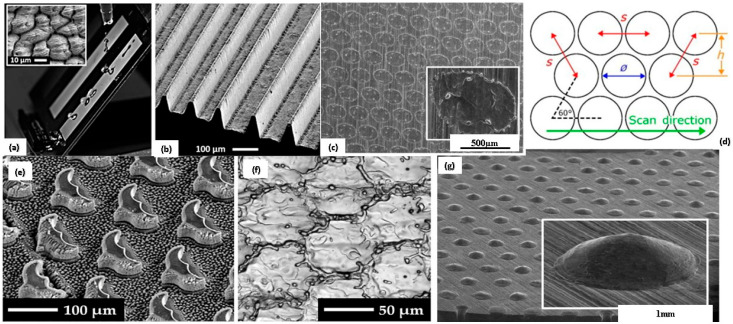
(**a**) Multi-scaled texturing for water-repellent surfaces by LST approach, (**b**) shark-skin-inspired structure texturing entails the reduction of skin drag friction to turbulence flow, (**c**) deep welding dots prepared by CW laser radiation, (**d**) Surface texturing by variable pulse energy and pulse overlap, (**e**) Surface texturing for enhancing boiling heat transfer, (**f**) formation of dimples for static friction enhancement, (**g**) Hemispherical surface textures formed by ns-pulsed LST [111,117,119]. Copyright Permission 2014, Elsevier.

Microscopic features over the surface texturing have huge potential for improving tribological properties by reducing friction. In addition to the reduction in friction, surface texturing can also be used to purposefully increase friction in a variety of applications that depend on friction to function properly. In recent trends, laser texturing has been used to enhance the tribological properties of the surface, leading to more attention toward the lubrication regime [108,109]. Surface texturing reduces the contact area and restores the wear debris for dry conditions [110]. The study depicted that multi-scale LST (laser surface texturing) imparted self-cleaning and water-repellent behavior over the surface, as shown in Figure 3a [111]. Besides that, the Rib-shaped structure inspired by the shark skin formed via the LST approach imparted a reduction in skin friction drag and wall shear stress on the solid surface in the turbulence condition, as shown in Figure 3b [111]. The focus for textured surfaces has been mainly on reducing friction, but few studies also showed that an increase in friction of the textured surface is also a beneficial aspect, correspondingly maintaining the low wear rate. Xiang et al. [112] analyzed the Al_2_O_3_/TiC composite textured with linear and zig-zag-like structures over the surface formed by the laser surface texturing approach, maintaining variable periodicity and similar width and depth. Regardless of groove periodicity, sliding speed, and geometry, texturing marked the enhancement in the coefficient of friction with a low wear rate. Conducive to friction, zig-zag surface texturing with low groove periodicity led to an increase in friction, attributed to the roughness of ceramic particles resulting in micro-cutting of edges of the groove [113]. Therefore, the entrapped wear debris over the surface of the groove entails a reduction in the wear rate. Similar behavior was observed with a few other materials, e.g., maskless electrochemical texturing over the steel surface as a working material entails higher friction in boundary lubrication, with 39% wear rate reduction (entrap of wear debris) [114,115,116]. Surface textures involving micro-holes, grooves, and dimples were effectively produced by LIPSS (Laser-induced periodic surface structure), as shown in Figure 3e,f [117]. The experimental analysis of various studies resembling the texturing of the surface by the LIPSS approach is depicted in Table 2. Other than LIPSS approach texturing, Wang et al. [118] formed the variable periodicity micro-grooves by femtosecond laser processing in steel, maintaining groove depth and width the same throughout. The outcomes revealed that the increase in COF with small periodicity was attributed to the reduction in restoring wear debris. Dunn et al. [119] formed a high friction surface, i.e., COF > 0.6, by varying the pulse energy and pulse overlap during surface texturing on steel depicted in Figure 3d and obtained the highest enhancement in friction by a factor of four with 0.8 mJ (pulse energy), and 50–95% (pulse overlap). More conclusive in detail, the enhancement in friction over the textured surface correlated with an improvement in surface hardness. Schille et al. [120] formed hemispherical texture and deep welding dots q-switched nanosecond and continuous wave (CW) laser processing on 42CrMo4 steel surface, as depicted in Figure 3c,g. Hemispherical texturing entails the enhancement in friction factor by 1.8, but for welding dots (diameter: 330 µm, height: 70 µm) increase in friction was decoded as 0.8. Hence, surface texturing is also a conducive aspect for increasing friction. 

The current review identified different bio-inspired surfaces playing a key role in tribological interaction in solid-solid and solid-liquid interaction resulting in exquisite behavior, i.e., anti-wear, drag reduction, self-cleaning, super-hydrophobicity, and reduction in friction. The current review aims to include studies investigating the tribological behavior of various species in nature. The primary focus has been to identify strategies used by these species to address tribological issues, which can lead to characteristics such as drag reduction, low friction, anti-wear, and anti-adhesion surfaces, which have been included in this review paper. The role of bioinspired surface morphologies in tribological applications is also discussed in detail. Surface texturing is paving the way toward creating low-friction surfaces, which has been covered in the current work. Various surface textures have been identified and listed in the paper, entailing the reduction as well as improvement in friction. The different studies have been discussed under sub-headings based upon examples from plants (mushroom-like structures, super-slippery surfaces, and tree-like bifurcation network texture) and animals (snake scale, sandfish, shark skin, scaly texture, oil-soluble additive, laminated structures, frog and cheetah) due to their difference in interaction with the surrounding Animals undergo significant motion as a results friction and wear becomes a prime concern. This review paves the way for development in the field of biomimetics, enhancing the tribological properties of the materials. The work-study mainly concentrates on providing a way to reduce friction. 

## 2. Biomimetic Surfaces Inspired by Animals 

**Figure 4:**
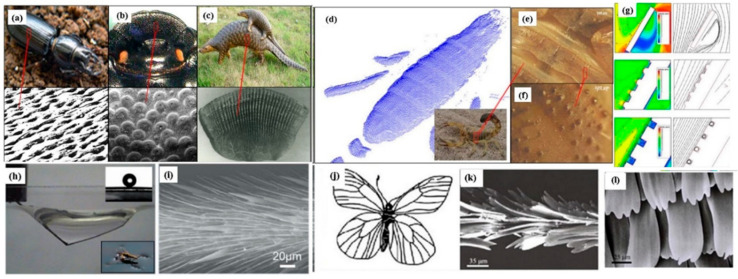
Surface texturing resemblance to (**a**) ground beetle, (**b**) dung beetle, and (**c**) Pangolin [131,152], Copyright permission from Elsevier, 2012 and 2001, (**d**) Scorpion surface (dorsal) obtained via laser scan, (**e**,**f**) scorpion back embedded convex hull and grove respectively, (**g**) anti-wear phenomena on scorpion surface via rotation of air over the groove channel enabling low speed-reverse flow zone [136], Copyright permission from ACS, 2012, (**h**) movement of Pond skater over the surface of the water, (**i**) SEM image of the movement of pond skater [148], (**j**–**l**) SEM image of surface morphology of butterfly wings and flat arrangement of scales over the butterfly wings [153]. Copyright permission from Elsevier, 2009.

The capability of the animals to survive in extremely harsh conditions has been enacted by the structural surface of the animal bodies [125]. The mimicking of surfaces for texturing inspired by different animals offers noise and drag reduction that develops anti-wear and anti-adhesion surfaces and enriches the surface in water-capturing ability. Wear (catastrophic failure) and tear of the surface of the animals surviving in the desert by the sandy wind make their life challenging [126,127,128]. Regardless of the survival of animals in the desert, wear is undesirable for many industrial applications reducing the lifespan of components and hindering the recycling ability of the components [129]. But the surface texture of the various animals, including the ground beetle, dung beetle, earthworm, mole cricket, centipede, ant, etc., prevents the soil from adhering to the surface of the animal’s body and restricts the soil wear [129,130,131]. Pertaining to marine biological applications, whelks and seashells comprising corrugated shells can effectively sustain in highly abrasive slurry environmental conditions [131,132]. Tian et al. [133] analyzed that the unequal lattice geometry of three typical shells in Ark Shells (Scapharca subcrenata) attributed to an excellent anti-wear characteristic. Tong et al. [134,135] analyzed that the micro-cracking and micro-shoveling attributed to the abrasive wear of different mollusk shells. Erosion is regarded as the major problem entailing the types of equipment failure and damage to the material, a phenomenon which is widely seen in, e.g., the nozzle of a rocket engine, helicopter rotors, turbine blades, and a few other mechanical parts/components [91,136]. There are few animals present in nature whose skin has evolved with erosion resistance, mainly including scorpions and desert lizards [137,138]. These animals can survive in the solid/gas mixed medium environment, i.e., sand, exhibiting high erosion resistance due to the biological functionality and unique surface texture/morphology [91,136,137,138]. Hang and Zang et al. [91,136,139] identified the anti-erosion functionality of the scorpion’s back and the outcomes of multi-coupling effects. Few research studies identified that surface morphology is one of the most critical factors in resisting erosion, i.e., the scorpion resists erosion without causing damage due to its surface morphology [136,140,141,142]. In some of the research studies, it was obtained that the scorpion body has a special arrangement of grooves on the back (evolution and adaptability to the living environment) that can alter the boundary layer flow over the surface and helps in resisting erosion [143,144]. Other than through erosion, few animals have roughness and hierarchical morphology over the surface that tends to impart superhydrophobicity (static contact angle > 150°) [143,144,145,146,147]. The strider is one of them, which has the ability to walk and stand on the surface of the water without getting wet [147]. The research group of Jiang and Gao [148] analyzed the structural morphology of the strider (especially the legs), covering the cuticle with wax and hairs with nano-grooves attributing to the superhydrophobicity of striders. Pertaining to superhydrophobicity, the surface morphology of a butterfly reveals the scales over the wings with overlapping edges that resemble roof tile morphology, promoting the directional super-hydrophobicity on a butterfly’s wings [149,150,151]. Figure 4a–l depicts the surface texture resembling ground beetle, dung beetle, pangolin, scorpion surface (dorsal) obtained via laser scan, scorpion back embedded convex hull, and grove, respectively. Anti-wear phenomena on the scorpion’s surface are provided by the rotation of air over the groove channel depicted in Figure 4g enabling a low-speed-reverse flow zone and movement of the pond skater over the surface of the water. Promoting the inherited advantage of the animal’s species (mobility, surface topography, skin) as per the surrounding response, the enacted surface morphology is desirable for anti-wear, anti-adhesion, and low-friction surfaces. Hence, more such animal species can be identified in creating these surfaces. 

**Figure 5:**
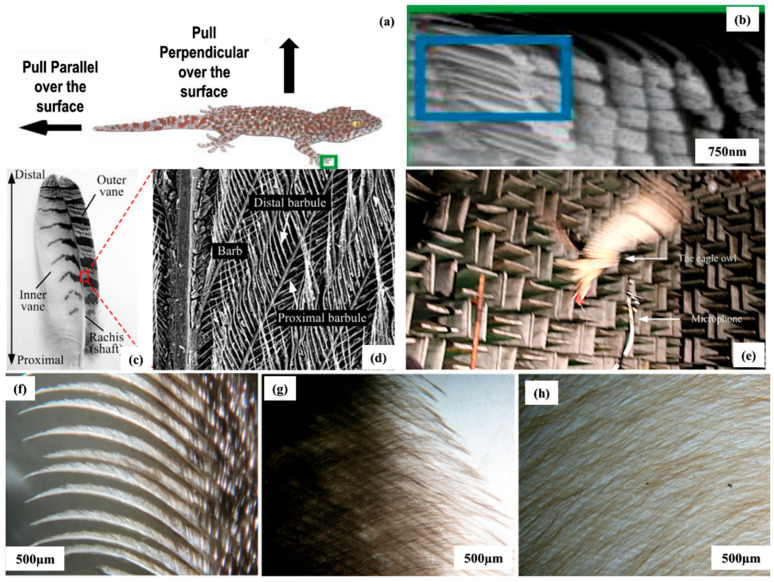
(**a**,**b**) Pictorial representation of Gecko along with SEM image of setae from the toe, (**c**–**e**) feather over the wings of owl in a different direction showcases the measurement of noise during flight, (**f**) feather at the leading edge, (**g**) trailing edge, and (**h**) surface of vane [128,173]. Copyright permission from Elsevier, 2012 and 2016.

For high adhesion (dry), Gecko is an eminent example that supports its weight and helps the gecko move against gravity [20]. This is possible due to the complex morphology (hierarchical) that inbuilt over the gecko toe and foot skin. The skin morphology entails branches, setae, spatula, and complex structures of fibrillar lamellae that are attributed to the attachment and detachment phenomena [154,155,156,157,158,159]. Further studies identified that the gecko has great adaptability toward surface roughness and acquired a larger surface area between the foot and the contact surface due to split ends [155,160]. Further investigation of the surface morphology of the gecko, it was identified that high adhesion was attributed to the adaptability and compliance of setae, as depicted in Figure 5a,b [161]. Since friction between the dry, hard, and macroscopic materials typically decreases during sliding and when velocity increases, friction continues to reduce due to the reduction in the interfacial contact [162]. However, the gecko setae did not exhibit a decrease in friction or adhesion while transitioning from static to kinetic contact mechanism [163]. Therefore, geckos have excellent stickiness due to millions of dry, hard setae on their toes. The requirement of a low adhesion against soil has been critically resolved by investigating the morphology of soil-burrowing animals, as they can move in the soil without any soil sticking over the body [164]. Other than gecko morphology, underwater animals are also suitable for drag reduction [126]. These include the surface morphology of underwater animals including sharks and carp [3,128,165]. The sector-like scaling surface of the carp, surrounded by micro-papillae attributed to superoleophilicity in water and air, serves the function of drag reduction [3,166]. Shark-skin surface morphology is another example of bioinspired structure attributing to the drag reduction over the surface. The surface of the shark skin is embedded with small individual tooth-like scales (dermal denticles) textured with some longitudinal grooves [167,168,169]. The presence of longitudinal grooves over the surface of shark skin allows them to align parallel to the flowing direction of the water. Along with flowing direction, a groove-like textured surface entails the reduction in the vortices formed over the surface (smooth), allowing effective movement over the surface of the water [170,171]. Resembling the morphological computation principle, the interaction between a non-smooth substrate and passive anisotropic scale-like material (shark skin) enhanced the locomotion efficiency of the robot walking on an inclined surface, resulting in low energy consumption. A significant example of scaling surface texturing is the Galapagos shark, whose surface provides a suitable reduction in the drag function [126,172]. Research studies depicted that some owl species can fly quietly, e.g., the eagle owl [128]. The surface morphology of the wings of an eagle owl involves feathers over wings, low flight noise (frequency), and low intensity of sound, which is suitable for sound absorption, as depicted in Figure 5c–h [173]. The feather structure over the wings (microscopic analysis) entails the enhancement in the fluctuation of the pressure around the turbulence boundary that causes a reduction in the vortex noise. To generate high friction forces on a wide range of substrates, the granular media friction pad (GMFP), inspired by the biological smooth attachment pads of cockroaches and grasshoppers, uses passive jamming [174]. The flexible membrane surrounding the pad’s granular media adapts to the substrate profile at contact [175]. The granular media passes through the jamming transition when under load, switching from fluid-like to solid-like characteristics [175]. High friction forces are produced on various substrate topographies by the jammed granular medium and the deformation of the encasing elastic membrane [174,175]. From the research studies, gecko surface morphology is highly recommended as a comparator when creating anti-adhesion surfaces, shark and carp surface morphology assists in obtaining drag reduction surfaces, and studying the wings of eagle owls provides a noise reduction advantage. 

### 2.1. Biomimicking Surfaces Inspired by Snake Scales and Sandfish

**Figure 6:**
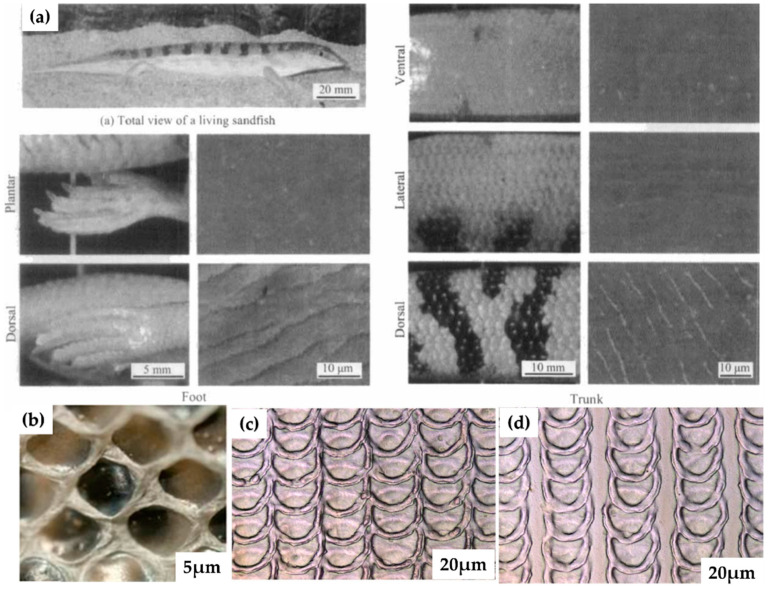
(**a**) Surface morphology of sandfish, (**b**) scales texturing attributed to the ball python, (**c**) vertical and horizontal overlapping of artificial scales over a snake (ball python), (**d**) only horizontal overlapping scales resembling snake skin [53,183]. Copyright permission from IOP, 2015; Copyright permission from Elsevier, 2015.

In the past decades, scientists identified the existence of life in the desert (harsh) conditions and evolved inspiration from nature, i.e., sandfish [176]. The evolution mechanism depicted that sandfish can move below the surface, revealing motion similar to swimming and dives into the sand [177]. Sandfish can swim at speed (10–30 cm/s) and move several centimeters laterally [178]. The scales over the surface of sandfish caused a reduction in the coefficient of friction (COF) [68,179]. The COF of the sandfish scale was observed to be better than PTFE particles, smooth and flat glass, steel (polished), and nylon surfaces (high density) [176,180]. As a result, the scaly texture of the sandfish hardly had any marks of wear when abrased against the sand. With a priority to reduce friction, the researcher identified that the arrangement and shape of the scale are vital in enhancing the tribological performance of the material surface [181,182]. Similarly, a group from Karlsruhe Institute of Technology mimicked two different surface textures (ball python and sandfish) on a bearing surface made of steel material and investigated tribological behavior under dry and lubricating regimes [53]. Figure 6 shows the scaling texture of sandfish and snakes. The outcomes revealed around a 40% reduction in the COF for the textured surface inspired by sandfish and a 22% reduction in the case of ball python when compared to the untextured surface. When mineral oil was used as a lubricant over the textured surfaces, three times further reduction was noticed for ball python texture and a 1.6 times reduction in sandfish texture than unlubricated condition [176]. From a future perspective, such surfaces can reduce COF in sensors embedded with lock brake systems (car), artificial hips, computer hardware, and machines running in a vacuum environment. It follows that the surface morphology of sandfish and snakes (ball pythons) are beneficial for research advancements in the field of tribology [53,176]. Considering the surface morphology of snake scales (hexagonal scales), the self-lubricating surface geometry acts as a prudent surface in creating a low-friction regime.

Furthermore, the ultra-low friction regime can be obtained with the snake scales by creating micro and nano-structures over the surface. Besides that, sandfish depicted no abrasion behavior under the sand, which helps extend the ultra-low friction regime. Sandfish morphology is quite a suitable inspiration when developing anti-wear surfaces. 

### 2.2. Biomimetic Surfaces (Shark Skin) Revealing the Riblet Effect

**Figure 7:**
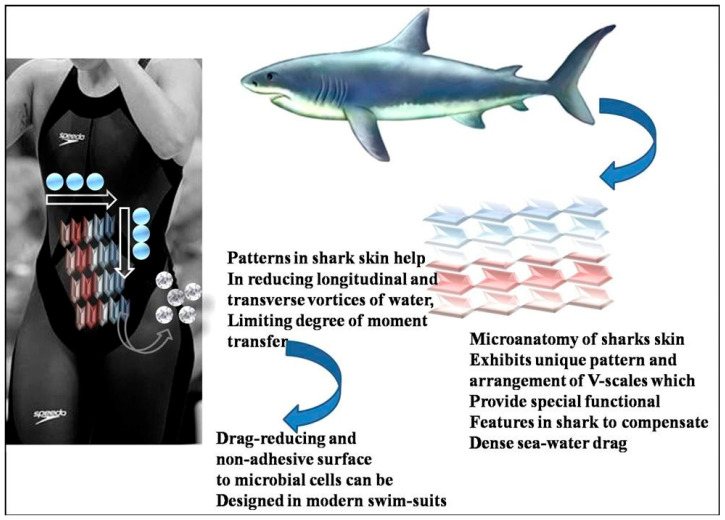
Shark skin’s low hydrodynamic surface drag is an inspiration for the design of high-performance swimwear with an antibacterial effect. The surface drag of water is significantly reduced by nature’s distinctive microscale design (Riblet effect). Arrows depict anti-microbial traits that resemble the micro-topography of a shark’s skin [192]—copyright permission from Elsevier, 2012.

The surface morphology of shark skin is the primary evidence of the riblet effect in bioinspired surfaces [25,46]. Riblet consists of fine structures embedded with consecutive grooves (longitudinal) [184]. Adhering to the microscopic scaling surface texture entails the free flowing of water into the grooves (shark skin surface) without the evidence of whirling [46]. This promotes the reduction in the drag force acting over the surface. Shark-skin surface textures over various materials have been mainly used in automotive, aircraft, and naval industry applications [185,186]. The formation of wing skin over the Airbus aircraft revealed the riblet effect, leading to drag reduction (6%) that entails fuel conservation [187]. Other than scaling texture, the new composite material (polyurethane) was also derived from the inherited surface morphology and characteristics of shark skin [188]. The usage of polyurethane composite material was seen in the BMW Z4 car model (hood, body components, and roof of the car), contributing to energy conservation [176]. Riblet effects were also utilized in the swimsuits made by Speedo International Limited [178]. In underwater photography, fabric and advanced design over the swim-suit trapped the air bubbles that kept the swim-suit dry; as a result, a reduction in water drag was observed as depicted in Figure 7 [189]. At the Beijing Olympics 2008, more than 60% of the swimmer used a speedo swim-suit embedded with the riblet effect, showcasing the new world record established by the swimmers [189]. The research studies indicated that the riblet structure and dermal denticles present on the shark surface were the reason for superior drag reduction, allowing fast swimming [184,190].

Furthermore, Miyazaki et al. [47] prepared a bioinspired riblet surface depicting the non-uniform morphology in shark denticles that paved the way for providing control over the local turbulent flow. The control over turbulent flow is suitable for fluid machinery and marine vehicle applications. Ibrahim et al. [52] developed the bio-inspired surface by shark denticles, paving the way for the enhancement in the marine vessel design (hydrodynamic) by macro-scale modifications in the hull design. Considering shark skin and denticle morphology, Lu et al. [191] prepared the bioinspired surface attributing to the reduction in water resistance. It is concluded that the micro-grooves over the skin allow the shark to move faster underwater, paving the way towards improvement in the performance of swimmers wearing bio-mimicked swim-suits contributing to drag reduction in water. Along with shark skin, shark denticles are also crucial in developing drag reduction. Mimicking the surface morphology of shark skin over the flexible surface can be considered a promising route toward obtaining more drag reduction over the surface. 

### 2.3. Biomimetics Surfaces Inspired by Scaly Texture

**Figure 8:**
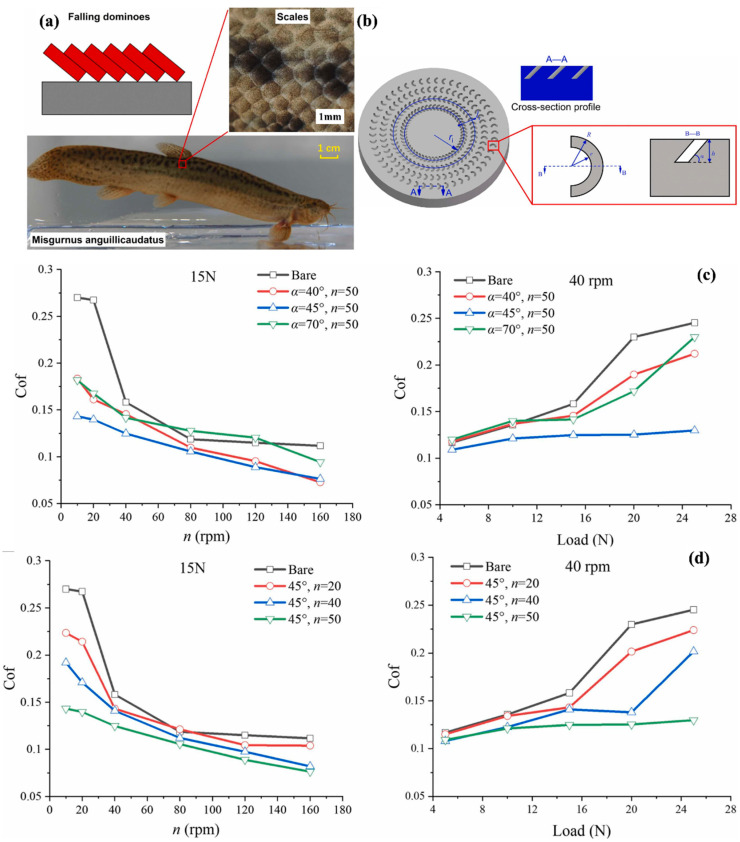
(**a**) loach’s scales are stacked up like falling dominoes, (**b**) geometrical characteristics of a bionic scaly texture and bionic scaly textures placed on a disc, (**c**,**d**) coefficient of friction was analyzed on the textured surface at different angles and different circumferential directions at a time, compared with the bare specimen [193]. Copyright permission from Elsevier, 2022.

Scaly structures inspired by pangolin and loach were used for surface texturing, reducing friction between solid/liquid surfaces and bio-surfaces [193]. As seen in Figure 8a, the loach scales fabricated through a micro 3D metal printer are stacked up like falling dominoes. They exude mucus as a lubricant to lessen wear and friction between their bio-surfaces and the solid or water they come into contact. The scaly surface textures on the metal surface were fabricated as depicted in Figure 8b in accordance with such stack-up structures and their lubricating capabilities. The geometric parameters of Figure 8b are provided in [193], and SLM (selective laser melting) approach was used to fabricate the textured surfaces [194]. Steel was used as the processing material in 3D metal printing [195]. For tribological testing, the specimen was fixed on the rotary platform (8 mm radius) for a time of 20 min, and white pharmaceutical oil was used as a lubricant [193]. The coefficient of friction was analyzed on the textured surface at different angles and circumferential directions at a time, compared with the bare specimen depicted in Figure 8c,d. Compared to standard textures with dimples or grooves, 3D structures with relatively deep layers allow more lubricant to be squeezed out and stored, magnifying the secondary lubrication effect to minimize wear and friction [196]. Therefore, the research studies concluded that under severe lubrication conditions of relatively low speed and high load, the effect of scaly textures on friction control was more substantial. The secondary lubrication amplification effect for textures with a reasonably high tilt angle close to 90° is comparatively mild [197]. Due to the deformation of the cantilever-beam-like structure of scaly textures with decreasing tilt angle, the deformation and contact stress concentration become increasingly substantial, leading to increased friction. The negative impacts of relatively high (70°) or low (40°) tilt angles may be countered by textures with a medium tilt angle (45°) [198]. Given the considerations above, textures with a medium tilt angle (45°) had a lower friction coefficient than those with high (70°) or low (40°) tilt angles. However, pre-lubrication suggests that lubricant in the sliding contact region is entrapped, which positively enhances the tribological behaviors of textured surfaces [193,198].

Meanwhile, to reduce the resistance in the water, a biomimetic surface resembling fish scales was formed at the surface of FKM (Binary fluorine rubber) by hot pressing at 150 °C followed by template replication using a 2800 mesh screen [199]. Self-cleaning and bouncing behavior was observed at the surface of the 2800FKM during the measurement of rolling and contact angle [199]. In comparison with FKM, the bio-inspired 2800FKM surface showed heat retention ability (98.89%) and contact angle (143.5°) along with self-cleaning behavior, illustrating the de-wetting performance. Under grease lubrication, no sign of wear was seen over the surfaces, and anti-friction and anti-wear behavior was observed over a 2800FKM surface in dry friction conditions [109,199]. Therefore, using the fish-scale structure for surface texturing over different materials is advisable to obtain superior tribological properties. The bio-inspired fish scale can aid the development of anti-wear surfaces. 

### 2.4. Bio-Inspired Green Dopamine Oil Soluble Additive

**Figure 9:**
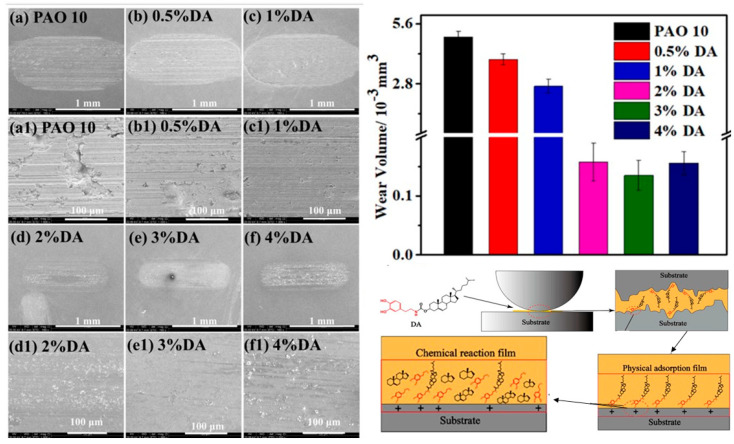
SEM morphology of wear-out sample with different compositions of DA in PAO 10 along with the critical value of wear rate. The 3% of DA in PAO 10 is the optimum/ideal value in terms of reduced friction and anti-wear surface [201]. Copyright permission from Elsevier, 2022.

To ensure the effective and long-term operation of the equipment, lubricants are used in mechanical equipment to lower the friction between the friction pair and reduce wear. Simultaneously, lubricating additives can dramatically increase lubricant performance. Most lubricant additives currently in use contain sulfur and phosphorus-based compounds and other highly ecotoxic elements, are poorly biodegradable, persistent in the environment, and quickly contaminate soil and water resources [200]. Therefore, researchers are keen to develop new ways of preventing environmental resources via green lubricating additives. There is a need of efficient green lubricant additives that are oil-soluble and have a good affinity to steel alloy, reducing friction, and improving anti-wear performance for steel/steel contacts. It has been demonstrated that the dopamine derivatives, a new class of chemicals based on amino and cholesterol hydroxyl modification, have high adhesion, oil solubility, and good lubricity [201]. All mammals, including dogs, have a pleasure center in their brain, which is stimulated by dopamine which entails a feeling of happiness [202]. The boundary adsorption and tribological performance can be improved by including the N element from dopamine in the additive molecule, which has the capability to adhere to various organic and inorganic surfaces [201,203]. Tribo-chemistry is essential for enhancing the effectiveness of lubrication as lubricants. At various concentrations of 0.5%, 1%, 2%, 3%, and 4%, samples of DA (Dopamine) were dissolved in PAO 10 (Poly alpha oil), as indicated in Figure 9 [201]. It was evident that DA has great oil solubility because it does not precipitate in PAO 10, and the oil sample remains clear [204,205]. Further investigations were performed into the synthetic DA’s physicochemical and tribological characteristics as a PAO 10 additive [201]. The viscosity and thermal stability of PAO 10 increase as DA content rises. In addition, as the DA concentration rises, PAO 10’s adsorption efficiency improves, and its contact angle with the metal surface decreases, demonstrating that DA has a strong affinity for the metal substrate and lowering PAO 10 surface energy at the metal interface. It enhances the tribological characteristics of PAO 10 and its lubricating efficacy, as indicated by Figure 9. Figure 9 also suggests that the concentration of 3% of DA in PAO 10 is the optimum/ideal value in terms of reduced friction and anti-wear surface. The 3% of DA in PAO 10 exhibits the best tribological performance compared to PAO 10 as displayed in Figure 9 [201]. Through electrostatic contact, the hydroxyl and amide bonds in the DA molecules first create a physical adsorption coating [206]. The friction pair’s surface is simultaneously reacted with by the active N and O components in the DA molecule, forming a protective tribo-film made of nitrate, cyanide, and iron oxide [201]. The tribological characteristics of PAO 10 are enhanced by this tribo-additional film’s obstruction of the friction pairs. As a PAO 10 lubrication additive, DA is essential, especially as a green lubricating additive that is being employed in obtaining low-friction surfaces.

### 2.5. Biomimetic Structures Inspired by a Laminated Structure

Layered materials have shown exquisite low-friction properties due to weak interlayer bonding [207,208,209]. The bio-inspired laminated structure from natural biological materials such as bones, shells, and spider silk can contribute excellent tribological properties [210,211]. Graphene is one of the most suitable bio-inspired materials that can be used to obtain laminated structures [212,213,214,215]. The nano-indentation and nano-scratch approaches were used to evaluate the tribological behavior of bio-inspired laminated aluminum matrix composite (BAMC) reinforced with graphene [216]. Compared to Al (pure), the friction resistance was improved by 28%, and the reduction in adhesion and ploughing was about 32% and 16%, respectively [216]. Upon nanoindentation on the biomimetic laminated structure, heterogeneous deformation at the interface of graphene intensified the strain hardening and improved the hardness, wear, and frictional resistance of BAMC. Nacre, often known as nature’s armor, has been used as a model for creating stronger and more durable bioinspired materials [217]. In nacre, hard aragonite bricks and soft biopolymer layers are arranged in a brick-and-mortar pattern by nature [217,218,219]. Despite knowing this, it has proven difficult to replicate all of the nacre’s reinforcing mechanisms in synthetic materials. To recreate the structure and reinforcing effects of nacre in aluminum composites, the hybrid graphene/Al_2_O_3_ platelets with surface nano-interlocks act as hard bricks for the main load bearer and mechanical interlocking and aluminum laminates as soft mortar [217]. The bioinspired graphene/Al_2_O_3_ doubly reinforced aluminum composite outperformed even nacre in terms of strength (223%), hardness (210%), stiffness (78%), and toughness (30%) when compared to aluminum. Along with mechanical properties, the tribology behavior was observed to improve in comparison with aluminum. The research study depicted that laminated ceramic materials are highly reliable in improving the tribological performance of the material surface [220,221,222]. Song et al. [223] investigated the friction behavior of Al_2_O_3_/MoS_2_-BaSO_4_ laminated material with reciprocating motion. The outcomes depicted a 20% to 40% reduction in friction as compared to the alumina. Hadad et al. [224] investigated the frictional properties of Si_3_N_4_-TiN laminated material and depicted no more improvement in the friction properties of laminated material. But the addition of hBN material to Si_3_N_4_-TiN laminated material entails less friction than Si_3_N_4_-TiN laminated material and Si_3_N_4_ material. From the research studies, it has been obtained that the layered materials offer improvement in the surface’s tribological aspect, inspired by bone, spider silk, and shells. Different composite materials formed with graphene as a matrix obtained better results than hybrid reinforcement. For future advancement, laminated structured material is a good idea for creating low-friction surfaces. 

### 2.6. Biomimetics Surfaces Inspirations for Improved Traction

**Figure 10:**
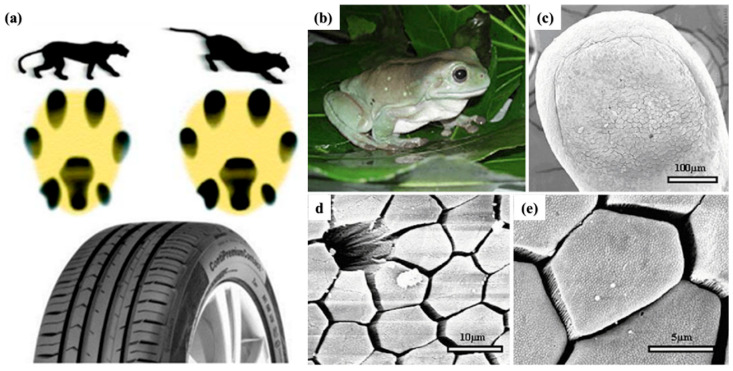
Biomimetics surface resembling the morphology of (**a**) cheetah paws inspired morphology, (**b**–**e**) tree frog morphology along with scanning electron microscopy of toe pad epithelium at a different scale. The micrographs: (**c**) pads of tree frog, (**d**) showing a mucous pore and (largely)hexagonal epithelial cells separated from each other at their distal ends by channels, and (**e**) indicates the presence of nanostructures on the ‘flat’ surface of the epithelial [176,234]. Copyright permission from Journal of Experimental Biology, 2016.

Strong traction between solids with rough surfaces occurs if at least one of the solids is soft (elastically). Meanwhile, some spiders and lizards can provide dry adhesion and move on vertical surfaces (rough) due to the presence of compliant layers present on the surface of their attachment pads [225]. Flies, grasshoppers, bugs, and tree frogs have less compliant layers on the surface of attachment pads, and the adhesion occurs on rough surfaces because the animals inject a wetting liquid into the pad–substrate contact area, generating a relatively long-range attractive interaction provided by the formation of capillary bridges [226]. On the other hand, the surface layer on the cheetah paws has more compliant layers, providing strong traction to the rough surface [227]. These surface morphologies of attachment pads and cheetah paws, providing strong traction, are quite beneficial for industrial applications as the variation in the morphologies provides a way to optimize energy distribution between the road and tires [176,178,226,227]. Considering that tires serve various purposes, including providing high sliding resistance during braking (stopping distance shorten) and low rolling resistance during driving (saving fuel consumption) [228,229]. During the formation of the tire bioinspired surface morphology inspired by the cheetah (characteristics), stalking the prey slowly and acquiring speed (high) for a short duration were incorporated [176]. The cheetah has flat barrow paws attributing to the low friction concerning ground contact during running, revealing low energy consumption as shown in Figure 10a. However, the flat paws broaden during the directional change in running and slow down the process, enhancing the contact area with the ground. Therefore, the transmission of force over a large surface area enhances stability. Thus, cheetah paws’ morphological changes are vital in optimizing stability across curve paths (high), effectiveness in direction change, and optimization of acceleration [230]. Resembling similar characteristics and traits, the summer tire Continental ContiPremiumContact™ was developed. The tire width was similar to conventional tires, but the morphology changes similar to cheetah paws; the tires’ width was widened during braking [178]. The widening of the tire width was attributed to the reduction in the stopping distance by around 10% to 12%. Therefore, the tire profile and selection of material used are critical in order to save energy consumption [178]. Another evidence of biomimetic surface attributing to energy consumption saving is the morphology of frog species [231]. The hexagonal pattern on the tire, which is similar to the frog species (tree frog and torrent frog), provides better performance (stopping distance reduction and grip optimization) to winter tires during wet conditions, as shown in Figure 10b–e [176]. Tree frogs (live on the tree, known for climbing) and torrent frogs (known to climb on wet surfaces near the waterfall) offer two bio-inspired surface morphologies resembling hexagonal patterns, suitable for the best performance in tires [232,233]. Other than that, the formation of the V-pattern tread of the tire is attributed to the evacuation (quick) of water from the contact surface, causing a reduction in the aquaplaning risks [176]. From the above studies, the morphology of cheetah paws and the hexagonal pattern on frog species are efficient in saving energy consumption. Widening the tire width contributed to a reduction in stopping distance. Therefore, varying the cheetah paws morphology will significantly impact saving energy consumption. Besides this, hexagonal patterns also act as a low-friction surface structure but depend on optimizing the parameters while mimicking the bio-inspired structure. Further, the micro-and nanostructures over the hexagonal patterns will produce better results in terms of providing improved tribological performance. Hence, these surface morphologies have developed anti-sticking surfaces. Table 3 further summarizes studies exploring the tribological behavior of various biomimetic surfaces and materials. 

## 3. Biomimetic Surfaces Inspired by Plants

In scientific theories and technical applications, such as self-cleaning, liquid-repelling, energy harvesting, and droplet manipulation that rely on reproducing the chemical properties and morphology of natural surfaces, a wide variety of biomimetic surfaces are provided by Plantae [237]. One well-known example of a liquid-repelling material is the lotus effect, which refers to the waterproofing properties of the sacred lotus plant’s surface [185]. Water rapidly beads up and rolls over the leaf due to the intrinsic benefits provided by the combination of hierarchical shape and wax-based chemical modifications, imparting a high contact angle with low adhesion and friction [238]. A few examples in nature also persist, which can trap water droplets that maintain high contact angle (essential for maintaining droplet shape), such as rose flower petals due to the high value of adhesion, known as the petal effect. This depends on the semi-wetting state of their hierarchical morphology between the Cassie-Baxter and Wenzel states [239]. However, these surfaces do not exhibit a persistent de-wetting condition and a potent repellency to liquids with low surface tension (Omni-repellency) [240]. The cuticles of springtails, a common arthropod, have been found to use another promising strategy to deflect most fluorinated fluids and maintain cutaneous respiration in quite moist conditions [241]. Such cuticles have given rise to morphological geometry that resembles a mushroom, ranging from singly re-entrant to triply re-entrant topology [242]. In addition to the static repellency, artificial liquid-repelling surfaces require repellency against impacting droplets, including minimal droplet-surface contact time to rebound droplets [243]. The normal behavior of droplets impinging on a super-repellent surface is for them to spread, retract, and bounce off with circular symmetry, resulting in the contact time bounded by an inertia-capillarity limit [237]. The symmetric spreading was avoided through interfacial features on macro curvatures. As a result, the asymmetrical droplet dynamics at impact have increased impalement resistance and decreased contact time. Recently, natural leaves and wings have inspired another strategy that abandons the conventional rigid prerequisite [237]. Flexible surfaces encourage kinetic repellency by minimizing impacting loads through their oscillations, extending the related research from statics to dynamics in contrary to the asymmetry process used by rigid surfaces [243]. The different bio-inspired surfaces have been enlisted in detail in the below section. 

### 3.1. Bio-Inspired Mushroom-like Structures

**Figure 11:**
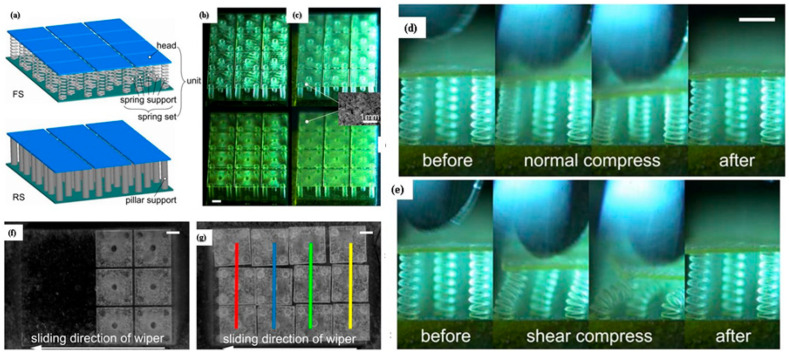
(**a**) Design of flexible and rigid singly re-entrant mushroom-like structure, (**b**,**c**) optical microstructure of corresponding surfaces using 3-D projection micro-stereolithography, (**d**,**e**) optical microscopy entails the mechanical robustness of mushroom-like flexible units against normal and shear compress, and (**f**,**g**) mechanical robustness of mushroom-like units against rigid and flexible surface after friction under 4 N (vertical lines resembling the similar behavior of FS and RS). Therefore, mushroom-like flexible structures are preferable for better tribology properties [237]. Copyright permission from ACS, 2021.

Through the use of three-dimensional projection micro stereolithography, a water-repellent biomimetic surface was created with single re-entrant mushroom-like basic units, each of which included a mesoscale head and a microscale spring set [244]. The research study showed that a single re-entrant mushroom-like structure repels the impinging droplets from the surface; therefore, FS (flexible surfaces) with low-energy particles combined to couple chemical modification to provide the kinetic repellency during the impact condition [245]. The mushroom-like flexible structure deformed in a downward direction when the head was compressed in a normal direction but came to its original state when the load was released [246]. The mushroom-like structure showed recovery capacity even when the head was under shear loading, suggesting good mechanical robustness resembling flexible support in relation to shear and normal compression, as shown in Figure 11 [247]. The tribology behavior of the mushroom-like flexible structure was studied for mechanical robustness at a normal load of (1, 2, or 4 N) with a speed of 1 mm/s, compared with rough surfaces (RS) [237]. The structural damage influenced coefficient of friction as a function of load. In the case of 4 N, the coefficient of friction first reached zero, but this was not the case in 1 and 2 N. The structural damage entails head fragmentations and breakages at pillar-bottom connections. Under the 4 N condition, on heads with irregular patterns, fragmentation was not directly seen, but by shearing the heads with a tweezer, some breakages at spring-head connections could be identified. Nonetheless, the ratio of damaged units on the FS was lower than that on the RS, even though both the FS and RS began to show structural damage with the same typical load of 4 N [237]. A previous research study depicted the structural damage at 0.04 N/mm, but a mushroom-like flexible structure can withstand higher loads without failure till normal load of 0.44 N/mm as well as high recovery potential in response to widespread normal and shear compression, indicating better mechanical robustness against tribological friction to approach real-world applications [248]. In terms of impalement barrier enhancement and contact time reduction, it is demonstrated that the flexibility of underlying spring sets improves the kinetic repellency of droplet infiltration, partially improving by 80% via structural tilting movements [242]. The flexibility gradient that results from incorporating different flexibilities in each mushroom-shaped unit was demonstrated to manipulate droplets directionally, thereby opening the door for droplet transport [237]. This is the primary example of using flexible interfacial structures that can effectively lower friction and improve water repellency. Flexible surfaces with low-energy particles provide kinetic repellency during the impact condition and can withstand without failure at a normal load of 0.44 N/m. Therefore, the development of flexible structures will be considered a suitable option for obtaining an improvement in the tribological behavior of a surface. 

### 3.2. Biomimetic Tree-like Bifurcation Network Texture

The tribological characteristics of biomimetic tree-like network texture, together with the liquid spreading flow properties, were analyzed in order to secure and extend the service life of the titanium alloy/ultrahigh molecular weight polyethylene artificial joint [249]. Three different surface textures (cross-shaped network, T-shaped network, and Y-shaped network) were created with various branch numbers and branch angles [249,250,251]. The texture ratios for each type of tree-like network are 10%, 15%, and 20%, respectively [252]. All three types of textured surfaces have high anti-friction characteristics and can minimize the immediate contact angle and achieve complete liquid spreading within a specific time frame [249,250]. The Y-shaped network texture exhibits the best liquid spreading flow property, whose instantaneous contact angle for a 2L liquid in a 10% texture ratio is 23°, and whose liquid may spread completely in 0.95 s [250,253]. In a 15% texture ratio, the T-shaped network texture’s friction coefficient drops to 0.077, a 38% reduction from the original surface [250]. Self-lubricating artificial joints benefit from this strategy for friction reduction [254,255,256,257]. Therefore, the Y-shaped network has been recommended to improve friction/tribological performance. The cross-linked network texture can find suitable resemblance in future advancements in the tribological aspects of the different bioinspired surfaces. Besides, surface texturing is always considered a beneficial aspect of improving the tribological aspects of the surface, depending upon the application usage. Furthermore, bifurcation network textures can be mimicked over the surface, resembling the morphological advantage of a Y-shaped network texture.

### 3.3. Plant-Based Super Slippery Surfaces

**Figure 12:**
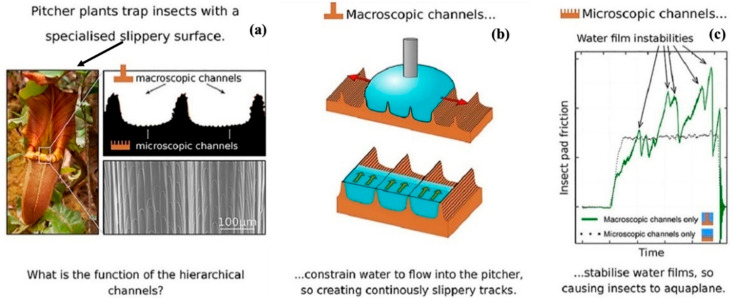
(**a**) Bioinspired pitcher plant surface paving the way for slippery surface, highlighting the micro- and macroscopic channels over the surfaces, (**b**) macroscopic channels allowing the flow of water into the pitcher plant, providing a slippery pathway, and (**c**) microscopic channels stabilizing water films and trapping insects [286]. Copyright permission from Elsevier, 2021.

**Figure 13:**
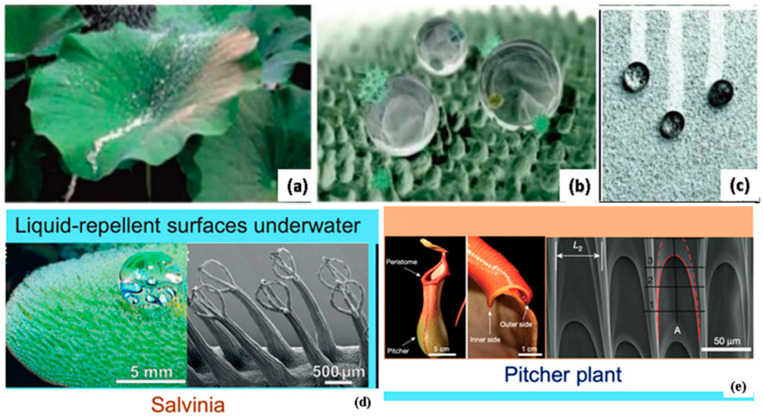
Biomimetics surface resembling the morphology of (**a**,**b**) normal lotus leaf and computer graphics embedded lotus leaf, (**c**) self-cleaning phenomena on painted surface mimicking lotus effect, (**d**,**e**) air-infused liquid repellent surface of Salvinia and the pitcher plant [176,283]. Copyright permission from ACS, 2022.

Typically, a light coating of lubricant with low surface energy involving fluorinated oil is applied to textured surfaces, creating slippery surfaces, often referred to as lubricant-infused surfaces [258,259]. Fluorinated materials are mainly used to infuse lubricants that produce a low-friction condition over the surface [260]. Although lubricant-infused surfaces are smooth enough for liquids to slide with a low contact angle hysteresis, liquids — including water and organic solvents — may not necessarily offer a significant contact angle on these surfaces [261]. These slippery surfaces use micro-textures to retain the lubricants, as a result, the lubricant layer repels the liquid, as opposed to surfaces that use reentrant shapes [262]. Liquid-repellent surfaces contain omniphobic surfaces and lubricant-infused slippery surfaces [263]. Pitcher plants are the perfect example of super-slippery surfaces that helps in producing low-friction surfaces. The pitcher plant, where insects slip into the pitcher and are then digested for food, served as the inspiration for the slippery surfaces depicted in Figure 12 [264]. Figure 12 shows the microscopic and macroscopic grooves over the surface, which are separated by ridges, providing hindrance to the lateral spread of water by enhancing the radial spread towards creating slippery surfaces [265,266]. A new class of liquid-repellent surfaces with self-cleaning capabilities can be created by the surface morphology of pitcher plants along with the microscopic and macroscopic grooves [267]. The pitcher plant surface has inherited a thin coating of lubricant, with surface features exhibiting natural durability both chemically and mechanically [268]. These slippery surfaces are used for fouling-resistant coatings and fluid-handling equipment in harsh environments [269]. These slippery surfaces paved the way for the improvement in the tribological properties of the surface [270]. Another example in this category is the lotus plant, regarded as an emblem of purity. The lotus plant (leaf) has unique characteristics, i.e., it does not get dirty and wet when exposed to rain and dust [271]. The droplet rolls over the surface of the lotus plant picking up the dirt, and keeping the leaves clean and dry after the rain, as depicted in Figure 13a–c. This happens as the adhesion between the dust and water particles is greater than the adhesion between the leaf and dust surfaces [272]. In the same regard, Barthlott et al. [273] evaluated the self-cleaning characteristics of the lotus. Further analysis at a higher magnification of lotus-inspired surface showed dust particle accumulation only at the asperity peaks [274,275,276]. Therefore, when the droplets fall over the surfaces, the dust particle gets washed away, demonstrating the self-cleaning behavior of the lotus leaf as depicted in Figure 13c [176,178,275,276]. Figure 13 depicts the biomimetic surfaces resembling the morphology of a lotus leaf and a computer graphic-embedded lotus leaf, as well as self-cleaning phenomena on painted surfaces mimicking the lotus effect. In the case of a superhydrophobic surface, the extreme water-repellent state is formed owing to the effective entrapment of air, contributing to lower friction and adhesion. Lu et al. [277] mimicked the surface morphology inspired by silver ragwort and lotus leaf to develop the fibrous mats pertaining the superhydrophobicity. The outcomes entail the presence of stable superhydrophobicity with a contact angle of 160° with lotus leaf and 147° with silver ragwort leaf. Other than lotus and silver ragwort leaf, pitcher plant-inspired surfaces are mainly effective in marine applications providing low adhesion can be seen in the reference [278,279,280]. Wang et al. [281] developed SLIPS aluminum, which mimics the pitcher plant with the anti-biofouling property of its surface, suitable for marine applications. Furthermore, research studies reveal that higher regularity and lower length provide suitable low adhesion properties at the surface, with higher SLIPS stability [269,281,282]. Since various biological surfaces have inspired the design of robust, air-resistant surfaces [283]. The lotus leaves, pitcher plant, and Salvinia are air-infused liquid-repellent surfaces [283]. All these use rough surfaces to trap air pockets. In the air, the layered roughness combined with the surface’s hydrophobic wax provides a stable and durable air-filled repellent layer for the lotus leaf [284,285]. The air layer can also be preserved or replenished during the transition from the air to the water environment [283]. For example, Salvinia uses hydrophilic patches on a superhydrophobic whisk-like substrate to strongly anchor the air-water interface, which helps establish the low friction and adhesion surfaces as depicted in Figure 13d. Figure 13e shows another air-infused liquid-repellent surface that has been discussed above. The surface morphology of pitcher-plant and Salvinia is ideal for developing slippery surfaces, while lotus, taro, and rice leaf create anti-wetting surfaces. Table 4 depicts the studies of various bioinspired surfaces drawing upon plants.

## 4. Conclusions and Future Outlook

The present work focuses on understanding different strategies devised by nature for modulating tribological interaction with the surrounding. Different tribological scenarios involving solid-solid, solid-liquid, and liquid-liquid interactions are discussed for reduced friction, adhesion, and wear. The water-repellant superhydrophobicity possesses low adhesion and friction, leading to exceptional properties such as self-cleaning, anti-fouling, and helps in drag reduction in submarines and vessels. The various examples of bioinspired surfaces are discussed that entail the modulation in friction and adhesion, i.e., lotus leaf surface (water repellency), gecko feet (directional-adhesion), micro-groove over shark skin (fast-swimming), eagle owl wings (noise reduction), snake scales and lizards (low-friction surfaces), sandfish bodies (high-wear resistance), and the pitcher plant and Salvinia (Super-slippery surface). Furthermore, the evidence of drag reduction was observed by mimicking the turtle’s surface morphology. The work study revealed that the bio-inspired approaches with tailored stiffness showed better outcomes in terms of low frictional properties. The development of micro-grooves inspired from shark skin through surface texturing can significantly minimize friction. The flexible mushroom-inspired surface was able to withstand high mechanical load without a failure than rigid surface. It also showed high recovery potential in response to widespread normal and shear compression, indicating better mechanical robustness along with improved kinetic impalement resistance. The multi-scale laser surface texturing is considered a suitable approach for imparting self-cleaning and water-repellent behavior over the surface. The straight and zig-zag-like structures over the surface formed by the laser surface texturing with variable periodicity, width and depth have been widely explored. The laminated/layered structures were also identified in the current study leading to the formation of low-friction surfaces. Graphene and similar 2D materials are suitable bio-inspired materials to obtain laminated structures.

From the perspective of future study, an ultra-low friction regime can be obtained by considering the surface morphology of sandfish skin which is renowned for its low friction and high resistance to wear against sand. In the same regard, the surface morphology of scorpions and tamarisk can withstand sand erosion exceptionally and are an appropriate inspiration for ultra-low wear surfaces. Since future advancements are more related to reducing energy consumption, ultra-low friction surfaces are beneficial in serving the same purpose. Furthermore, surface texturing inculcating dimples, grooves, or convex on surfaces of friction units using mechanical or chemical processing technologies are attracting the research trend towards improvement in tribological performance. The surface texture of the animals, including ground beetle, dung beetle, earthworm, mole cricket, centipede, ant, etc., restricts the soil wear and can be further explored. In the same context, pangolin and loach inspired structures are discussed in this review article that helps reduces the friction between solid/liquid surfaces and bio-surfaces. More particularly, the flexible interfacial structures can effectively resist tribological friction and encourage water repellency. Bio-inspired hierarchical structures should be considered for the development of low-friction surfaces. The surface morphology of the snake scale can inspire the development of surfaces with directional friction properties. The overlapping scales of the snake’s skin with protrusions in the shape of teeth help control wear and friction. However, such surfaces have not been explicitly explored on metal surfaces. The possibility of utilizing snake-inspired textures for reducing friction and wear and how these surfaces perform in the presence of lubricants can be evaluated. Although reports of hierarchical patterns’ favorable impacts on adhesion and friction have been observed, the impact of the pitch of nano-scale features has not been thoroughly studied. Creating efficient green lubricant additives that are oil-soluble, have a good affinity towards steel for reducing friction, and improve anti-wear performance for steel/steel contacts is attracting research attention.

## Figures and Tables

**Table 1:** Biomimetic surfaces used for drag reduction.

S. No	Researcher	Surface Topography	Research Outcomes	References
1	Wu et al.	Fish Scales attributing to water trapping	biomimetic surface resembling water-trapping fish scale microstructures is effective in reducing drag resistance. The outcomes revealed the drag reduction is around 2.085%.	[34]
2	Domel et al.	Denticles	The object was the flow rate and denticle size, and the hydrodynamic characteristics of 3D-printed shark-skin foils were investigated. The outcomes revealed the drag reduction is around 35%.	[35]
3	Heidarian et al.	Riblet	The impact of various riblet types was examined using computational fluid dynamics. The outcomes revealed the drag reduction is around 11%.	[36]
4	Song et al.	Barchan dunes	To research the impact of drag reduction, a planned and simulated non-smooth surface with barchan dunes-like contours was developed. The outcomes revealed the drag reduction is around 33.63%.	[37]
5	Wen et al.	Denticles	A flexible, synthetic shark skin membrane was created and put to the test in the water. The outcomes revealed the drag reduction is around 5.9%.	[38]
6	Han et al.	Denticles	In a water tunnel, a biomimetic surface created via the exact duplication of shark skin was put to the test. The outcomes revealed the drag reduction is around 8.25%.	[39]
7	Rastegari et al.	Riblets	DNS (direct numerical simulation) looked at the general mechanism of superhydrophobic longitudinal microgrooves and riblets reducing turbulent drag. The outcomes revealed the drag reduction is around 61%.	[40]
8	Khan et al.	Dragonfly	Experimental evaluation using the 3D printer in a wind tunnel at different angles and speeds. The outcomes revealed that the higher angle and low speed entail a suitable drag reduction.	[41]
9	Arunvinthan et al.	Shark scales	The vortex model resembled shark scales and was applied to NACA 0015 airfoil that revealed the reduction in drag	[42]
10	Yakkundi et al.	Rear wings spoiler	Automobile models with rear wings spoiler were obtained at 70 km/h and depicted a drag reduction of around 8.2%.	[43]
11	Kim et al.	Golf ball embedded with no dimples but tiny grooves	Measured the drag coefficient over the gold ball embedded with tiny grooves and obtained that the drag coefficient of the micro-groove surface was higher as compared to dimple surfaces.	[44]
12	Kozlov et al.	Box-fish	Analyze and compute the computational behavior of box fish-inspired texture for drag reduction. The outcomes depicted that the bluff geometry in the case of box fish has obtained the most appropriate drag reduction.	[45]
13	Chen et al.	Shark Skin	Anti-fouling is regarded as the most suitable for drag reduction. The experimentation uses shark skin morphologies for surface alteration.	[46]
14	Miyazaki et al.	Riblets	The experimental study entails the biomimetic riblet inspired by the bumps of shark skin with round pattern grooves and rough surfaces over the bumps.	[47]
15	Dia et al.	Shark-skin	Shark-skin orientation illustrates the ridge and flow direction entails the fluid behavior effect on the surface of the water. The outcomes depicted by the rheometer entail that the uniform particles have a minimum velocity gradient at a 90° angle (orientation).	[48]
16	Sen et al.	Vortex	Several types of vortex generators were studied and drag reduction was evaluated for the different vortex generators.	[49]
17	Wen et al.	Shark-skin	Modification in the spacing and the arrangement of bumps (shark skin)	[50]
18	Mutukumar et al.	Fish skin	In the experiment, a collection of fish skin acted as a transition to the turbulent boundary layer and formed an overlapping of the fish array structure. The outcomes revealed a 27% drag reduction was observed.	[51]
19	Ibrahim et al.	Riblets	Riblets motivated by shark skin denticles subtended to the change in the marine vessel’s structures. The outcomes revealed that a 3.75% reduction in drag was observed.	[52]

**Table 2:** Surface texturing formed by LIPSS approach.

S. No	Material Used	Texturing Type	Periodicity (nm) and Depth (nm)	Tribo-Test and Sliding Direction	Increase in Frictional Factor	References
1.	100Cr6 steel	Periodic Groove	90 nm; 200 ± 30 nm	Ball on disk type test and direction is Perpendicular to LIPSS	Maximum 4	[121]
2.	Co-Cr-Mo alloy	Single and multi-scale groove	800 nm	Ball on disk type test and direction is Perpendicular to LIPSS	Maximum 3	[122]
3.	Single Silicon (p-doped) Crystal	Periodic Groove	730 nm; 230 nm	Ball on disk type test and direction is Perpendicular to LIPSS	Maximum 3.5	[123]
4.	Crystalline Silicon	Periodic Groove	750 nm; 150 ± 50 nm	Ball on disk type test and direction is Perpendicular to LIPSS	Maximum 1.6	[124]

**Table 3:** Study of different bio-inspired textures and materials for tribological properties.

S. No	Researchers	Bioinspired Texture/Material	Experimental Evaluation/Outcomes	References
1.	Chaoyang et al.	Dopamine	Excellent tribological characteristics are exhibited by bioinspired Dopamine (DA). The best tribological properties are seen when the DA concentration in PAO 10 approaches 3%.	[201]
2.	Zehua et al.	Fish inspired texture	Outstanding heat retention rates (98.89%), excellent wetting performance (Contact angle = 143.51°), and self-cleaning are all features of 2800FKM.	[199]
3.	Yang et al.	Loach and pangolin scaly texture	Friction between bio-surfaces and their contracted solid/water is decreased by a scaly surface.	[193]
4.	Junya et al.	Surface modification by bio-inspired nanoparticles	To improve interfacial adhesion, polyethyleneimine (PEI), dopamine (DA), and SiO_2_ nanoparticles were co-deposited onto the surface of the Basalt/PTFE fabric. To improve the tribological performance of fabric composites, CaF_2_ and Si_3_N_4_ were added to fabric composites	[235]
5.	Tramsen et al.	Granular Media friction pad inspired by cockroach and grasshopper	When a load is applied, the granular medium goes through the jamming transition, changing its properties from fluid to solid. High friction forces are produced on a variety of substrate topographies by the jammed granular medium in conjunction with the deformation of the encasing elastic membrane.	[174]
6.	Yi et al.	Colloidal hydrogel system of aluminum hydroxide nanosheets (ANHS)	Colloidal hydrogel develops excellent stiffness and elasticity, as seen by its elastic modulus of >10 MPa. It has been shown that AHNS hydrogel works well as a lubricant and an anti-corrosive.	[236]
7.	Tian et al.	Ark Shells	Unequal lattice geometry of three typical shells in Ark Shells (Scapharca subcrenata) attributed to an excellent anti-wear characteristic	[133]
8.	Hang and Zang et al.	Scorpian back	The outcomes depicted the anti-erosion functionality of scorpion back	[91,136,139]
9	Xiang et al.	Straight and Zig-Zag Texturing	Al_2_O_3_/TiC composite textured with straight and zig-zag-like structures over the surface formed by the laser surface texturing approach, maintaining variable periodicity and similar width and depth. Regardless of groove periodicity, sliding speed, and geometry, texturing marked the enhancement in the coefficient of friction with a low wear rate.	[112]
10.	Tong et al.	Mollusk shells	The micro-cracking and micro-shoveling attributed to the abrasive wear of different mollusk shell	[134,135]

**Table 4:** The tribological behavior of various biomimetics surfaces by plants.

S. No	Researcher	Bio-Inspired Texture	Experimentation/Outcomes	References
1.	Liu et al.	Lotus leaf	The presence of stable superhydrophobicity with a contact angle of 160°	[277]
2.	Klicova et al.	Lotus leaf	Anti-adhesion surfaces of nanofibrous mats provide low adhesion inspired by the lotus leaf.	[287]
3.	Hu et al.	Flexible Mushroom structure	Structural damage at 0.04 N/mm but a mushroom-like flexible structure can withstand without a failure at a normal load of 0.44 N/mm as well as high recovery potential in response to widespread normal and shear compression, indicating better mechanical robustness against tribological friction to approach real-world applications	[237]
4.	Liu et al.	Silver ragwort leaf	Presence of stable superhydrophobicity with a contact angle found to be 147° with silver ragwort leaf	[277]
5.	Barthlott et al.	Lotus leaf	Examine the self-cleaning characteristics of the lotus, developing a color façade StoLotusan, revealing a similar surface morphology to that observed in the lotus leaf.	[273]
6.	Song et al.	Lotus, marigolds, and red rose	Superhydrophobic copper meshes were developed and prepared, followed by etching and modification with 1-dodecanethiol over the surface. The resultant copper foam removes organic solvents below and above water. The 153° ± 3° was the contact angle (static) obtained. Hence, this enhances copper cloth, a good tool for oil spill cleanup as well as oily wastewater treatment.	[288]
7.	Li et al.	Lotus and pitcher plant	Transformable liquid-resistant fabric surfaces were formed using a simple one-pot approach. The surface of PDMS@Fe_3_O_4_ fabric was formed, with lotus leaf-like characteristics retaining slipperiness over the surface. Other than that, the lubricant-infused surface with a continuous coating resembles the rim of a pitcher plant.	[289]
8.	Jiang et al.	Cactus spine	Fog collection characteristics of cluster-distributed trichomes and their surface structural characteristics were discovered.	[290]
9.	Labonte et al.	Pitcher plant	The study concluded that bioinspired surfaces from pitcher plants possess omni-repellent characteristics on the surface that grant non-stickiness nature to the surface. Neither polar nor non-polar liquids would stick on the surface.	[286]

## Data Availability

Not applicable.
